# Promoting interdisciplinary research to respond to public health crises: The response of the Columbia University CTSA to the opioid crisis

**DOI:** 10.1017/cts.2019.426

**Published:** 2019-10-30

**Authors:** Jennifer L. Humensky, Zainab Abedin, Kawthar Muhammad, Michelle McClave, Tiara Torres, Elisabeth Swift DiMaria, Muredach P. Reilly, Harold Alan Pincus

**Affiliations:** 1 Irving Institute for Clinical and Translational Research, Columbia University Irving Medical Center, New York, NY, USA; 2 New York State Psychiatric Institute, New York, NY, USA; 3 Division of Cardiology, Department of Medicine, Columbia University Irving Medical Center, New York, NY, USA

**Keywords:** Opioids, CTSA, interdisciplinary, public health, public policy

## Abstract

Effectively addressing public health crises requires dynamic and nimble interdisciplinary collaborations across the translational spectrum, from bench to clinic to community. The Clinical and Translational Science Award (CTSA) Program hubs are uniquely suited to facilitate interdisciplinary collaborations across universities and academic medical centers. This paper describes the activities at the Columbia University CTSA Program hub to address a current public health crisis, the opioid epidemic. Columbia’s CTSA Program hub led a three-phase approach, based on the Conceptual Model of Transdisciplinary Scientific Collaboration as described by Stokols *et al*.: (1) a university-wide planning and brainstorming phase to identify key leaders across many domains who are influential in addressing the opioid epidemic, (2) a campus-wide and community outreach to identify all interested parties, and (3) ongoing targeted support for collaboration development. Preliminary metrics of success are interdisciplinary collaborations and grant funding. We describe recent examples of how interdisciplinary collaboration, academic-community partnership, and pilot funding contributed to the development and funding of innovative interdisciplinary research, including the New York site of the HEALing Communities initiative. The processes are now being used to support interdisciplinary approaches for other translational public health issues.

## Introduction

The opioid crisis in the USA shows no signs of abating. In 2015, over 33,000 people died from opioid overdoses. By 2016, this had risen to over 42,000 [1]. Addressing the opioid crisis requires focusing on prevention of inappropriate opioid use, including development of new therapies for pain and treatment of addiction [2]. Inappropriate opioid use can stem from a number of factors, including underlying illnesses or injuries causing pain or addiction to substance use [3]. Thus, interdisciplinary collaboration is required to effectively address this crisis. For example, effective collaborations might include specialists treating the underlying disease (e.g. oncology), specialists treating injuries (e.g. orthopedics), primary care and emergency physicians treating individuals with chronic pain [4], addiction treatment specialists, community-based mental health specialists [5], criminal justice specialists [6], and informatics linking these systems together. Promising treatments have been developed when specialists work together to address the comprehensive issues.

However, due in part to the specialized training and focus of academic specialties, many researchers need assistance or support to build interdisciplinary collaborations [7]. The National Institute of Health (NIH) has codified the approach of addressing complex clinical problems through the collaboration of multiple disciplines as team science [8]. With investment in research funding, notably through the Helping to End Addiction Long-term (HEAL) initiative, the NIH is seeking to identify solutions that affect “every domain of family and community life” [9]. To effectively address this crisis, clinicians and researchers need to develop interdisciplinary collaborations, including an understanding of the policy, economic, and public health impacts of opioid misuse [10].

The Clinical and Translational Science Award (CTSA) Program is uniquely suited to foster interdisciplinary collaborations within and across universities and academic medical centers. CTSA Program hubs, supported by the NIH National Center for Advancing Translational Science (NCATS), are currently located at 58 academic medical centers across the USA, as of fiscal year 2018. CTSA Program hubs are designed with the hub-and-spoke model, with a central “hub” at each institution that facilitates interactions with “spokes” – departments, divisions, and schools across the university and medical center as well as regional partners including other hospitals, health clinics, and nonprofit organizations. These hubs provide resources that promote collaboration, including providing data and informatics core support and community engagement resources to help investigators identify partners and participants for clinical trials, and supporting researchers (e.g. through educational opportunities and pilot grant funding) that can facilitate collaborations. Additionally, CTSA Program hubs aim to help build connections with entities outside of the university/medical center, including with community-based nonprofit organizations and the pharmaceutical and informatics industry. The CTSA model of “Develop, Demonstrate and Disseminate” is well suited to help foster high-quality research, evaluate and demonstrate efficacy, and disseminate findings, beyond peer-reviewed publications, into materials usably by stakeholders such as community-based organizations and industry representatives.

This article describes how one CTSA Program hub, located at Columbia University’s Irving Institute for Clinical and Translational Research (Irving Institute), is responding to this public health crisis by organizing experts from numerous disciplines, both inside and outside of the university, to promote interdisciplinary dialogue, explore ways of designing research to address this crisis, and provide opportunities for collaboration across multiple entities.

## Methods

**Fig. 1.**
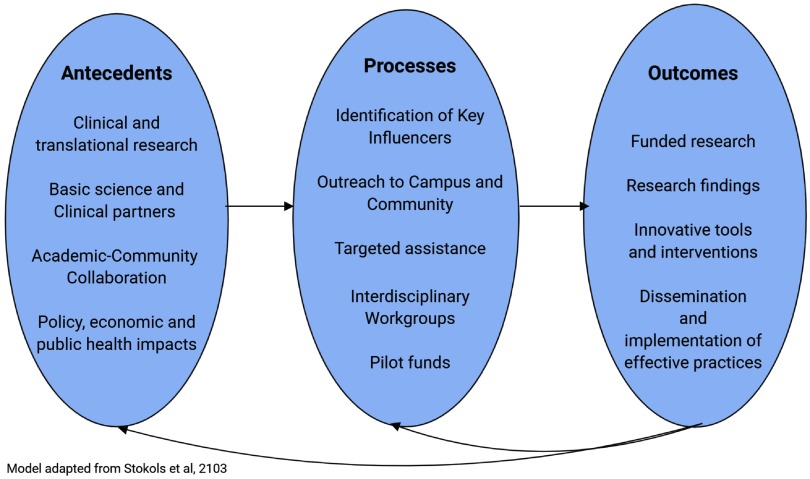
Conceptual model of Columbia University CTSA development of interdisciplinary scientific collaboration.

The Irving Institute’s opioid response is based on the conceptual model of transdisciplinary scientific collaboration, described by Stokols *et al*. [11], which involves identifying factors necessary for interdisciplinary collaboration, collaborative processes, and outcomes. The model was adapted so that the antecedents, processes, and outcomes fit the activities of the Irving Institute in promoting interdisciplinary translational research. Fig. 1 illustrates the conceptual model, including (a) identification of the need for collaborative research that utilize clinical and translational research, including collaboration between basic science, clinical partnerships, and community partnerships and (b) the development of a three-phase process to facilitating interdisciplinary collaborations: (1) a university-wide, planning and brainstorming phase to identify key leaders across many domains who would be influential in addressing the opioid epidemic, (2) a campus-wide and public outreach, to publicize efforts to all parties who would potentially be interested in joining this effort, and (3) ongoing targeted assistance and support through interdisciplinary work groups, match making, and seed funding for development of collaborations and funding applications. These activities are yielding fruitful collaborations resulting in fundable research that can help to prevent and treat opioid misuse. Institutional Review Board approval was not required for this project.

### Identification of Key Leaders

The first step in this process is identifying individuals who are key leaders across the broad range of disciplines with expertise in addressing the crisis. The initial planning phase began in the fall of 2017 with a local environmental scan, identifying relevant experts across the medical center and the university, for example, in the schools of Medicine, Dentistry, Nursing, Public Health, Arts and Sciences, Business and Engineering as well as in the hospital and community. This led to the convening of informal meetings, with participants identified via a snowball method – participants recruited other participants who were identified as having expertise in needed areas. The brainstorming phase culminated in a seminar attended by almost 100 participants on December 21, 2017, to identify how the Irving Institute could best support collaborative research to address the opioid crisis. Speakers at the seminar included representatives from across the university, including Psychiatry, Anesthesiology, Biomedical Engineering, and Medicine, as well as New York Presbyterian Hospital and the New York State Psychiatric Institute, and the upper Manhattan community covered topics, including the mechanisms of pain, epidemiology of addiction, and informatics needed to coordinate data sharing across multiple systems.

Following the plenary sessions, participants were invited to join one of the four breakout sessions: (1) improving prescribing and pain management, (2) improving addiction treatment, (3) engaging community partners and resources, and (4) facilitating bench to bedside discoveries and translation. Each breakout session generated recommendations for their respective focus areas to address the opioid crisis as well as potential additional participants in the process.

The four breakout sessions evolved into ongoing work groups, with 11–17 members each, incorporating expertise in each of the four content areas. The work groups continue to meet on a regular basis, to develop new collaborations and stimulate research within and across work groups and beyond Columbia, as well as engaging colleagues at other CTSA hubs.

### Campus-Wide and Public Outreach

Following the identification of key leaders, the next phase consisted of systematic efforts aimed at identifying and engaging all investigators who are interested in, or working in, an area relevant to the opioid crisis. Activities have included a campus- and community-wide interdisciplinary symposium, campus-wide email request, and ongoing monitoring of funded grants and published papers. The CTSA Program hub began planning an interdisciplinary symposium to discuss issues related to prevention and treatment of the opioid crisis, from January to June 2018. The symposium, held on June 22, 2018, on the campus of the Columbia University Irving Medical Center in New York City, included 192 registered attendees. Attendees represented a wide spectrum of departments and divisions from across Columbia University/New York Presbyterian Hospital, including (but not limited to) the schools of medicine, dental medicine, psychiatry, business, social work, and public health. In addition, attendees included representatives from federal, state and local government agencies, local community-based organizations, industry firms, and other local universities/academic medical centers.

The symposium included a plenary session in which speakers from the NIH describing funding priorities, including Michael Kurilla, Director of the Division of Clinical Innovation at the NIH NCATS and Carlos Blanco, Director of the Division of Epidemiology, Services and Prevention Research at the National Institute for Drug Abuse. Speakers also included experts in epidemiology and addiction treatment discussing the current state of research in the field, and New York City Commissioner of Health who discussed the City’s current activities. Following the plenary session, two breakout sessions were held – one focusing on pharmaceutical industry activities and one focusing on activities of local community-based organizations. The day also included a luncheon in which networking was facilitated through interdisciplinary-themed luncheon tables.

Following the symposium, we further aimed to identify as many investigators as possible with opioid-related research interests. Investigators have been identified through campus-wide email blast, as well as from visitors to our dedicated website. We also routinely conduct searches for additional NIH-funded investigators (though NIH Reporter) and opioid-related publications (through PubMed and Google Scholar) generated by Columbia-affiliated faculty.

### Ongoing Targeted Assistance and Support

Following the symposium, participants were invited to join one of the four specialized working groups (addiction, pain management, community resources, and bench to bedside). Each group identified between three and eight new members. The working groups continue to meet and collaborate on research and grant ideas.

Additionally, a Steering Committee was developed, comprising leaders from each of the four work groups and leaders in each of the specialized practice areas. The Steering Committee meets bimonthly and serves as an overall brain trust for the effort. Members continue to meet to discuss newly funded grants and upcoming grant applications and identify the need for collaborators. For example, an investigator might discuss the need for an economist on an upcoming grant application and solicit suggestions for economists who have worked in these areas.

The Columbia CTSA continues to promote communication by maintaining a database of interested investigators, establishing regular communications with and across work groups, convening steering committee meetings, and providing website updates on the initiative. The database (which currently has nearly 200 members) is used to distribute information on funding opportunities and new developments through an email listserv. It is also used for collaborator identification. For example, if an investigator contacts the CTSA program hub and asks for assistance in identifying collaborators working in the dental school, they can be put in touch with dental faculty who are included in the database. The CTSA program hub keeps track of opioid-related federal grants awarded and peer-reviewed publications from Columbia University and the affiliated New York State Psychiatric Institute; these listings can also be used to identify faculty working in these areas. We also connect investigators with other CTSA resources, including the Trial Innovation Network which provides resources for investigators developing clinical trials.

Information on Columbia CTSA program hub activities is maintained on a public website (https://www.irvinginstitute.columbia.edu/about-us/key-initiatives/opioid-crisis-response), with an email address to contact the team. The website provides search links which enable real-time searches of all federal and nonfederal funding opportunities with a keyword of opioid. Additionally, the CTSA Opioid response team collaborates with other external initiatives, including the National Academy of Medicine’s Collaborative on Encountering the US Opioid Epidemic. Table 1 summarizes the goals and examples of key activities over the duration of the opioid initiative.

**Table 1:** Steps for facilitating interdisciplinary collaboration

Activity	Examples
Identification of key leaders	Review funded grants, published papers to identify investigators working in this area, by using databases such as NIH Reporter and PubMed
	Through a snowball method, identify other investigators and potential partners
	Brainstorming meeting of key leaders to assess potential solutions
	Smaller working groups to address targeted issues
Large-scale (campus-wide and public) outreach	Symposium or other large meeting open to campus and community
	Establishing avenues for ongoing communication, for example, through email listserv, websites
	Establishment of smaller working groups
	Regular email blasts to university community to identify other interested investigators
Ongoing targeted assistance and support	Steering committee to set priorities
	Ongoing small working groups
	Regular publicity of funding opportunities
	Maintaining a database of interested researchers to facilitate introductions
	Facilitate pilot funding where possible to seed collaborative research

## Results

The success of this CTSA interdisciplinary intervention can be measured, on a preliminary basis, by (1) the success in supporting new interdisciplinary collaborations, (2) later, on securing additional grant funding for research, (3) with time, on the impact of CTSA activities on grant funding and publication, and (4) ultimately, on new research output (e.g. new discoveries in pain and addiction science or new pain therapies), implementations, and intervention that impact the opioid crisis. Table 2 provides some descriptive information about grants submitted by Columbia University researchers since the initiative began in September 2017. To date, 41 unique grants have been submitted by investigators who are affiliated with the initiative. As most of these grants are under review, detailed information cannot be shared. To date, 14 of the grants have been funded, 17 are still pending, and 10 were not funded. The 41 grants represent 21 Principal Investigators (PIs). The annual direct costs for NIH grants alone total $28 million (not counting grants from other federal agencies or nonfederal funding sources). While the largest number of PIs is in the Psychiatry department, PIs also came from a variety of departments and schools across campus, including Epidemiology, Health Policy and Management, Sociomedical Sciences, Anesthesiology, Orthopedic Surgery, and Social Work. In addition to stimulating development of New York’s HEALing Communities project (which is described further below), some examples of our CTSA facilitating interdisciplinary activities include:

- Two investigators (Rachel Shelton, ScD, Sociomedical Sciences and Lisa Rosen-Metsch, PhD, dean of Columbia’s School of General Studies) were awarded a series of CTSA pilot grants to advance understanding of how to implement opioid education and naloxone training on college campuses. As part of Phase I of the pilot, investigators collaborated with a multidisciplinary research group across the university, to establish a platform for implementing evidence-based practices and programs that address the opioid epidemic. A second CTSA pilot was funded to further support this work on a larger scale, which will examine the acceptability, feasibility, and implementation of opioid awareness education and naloxone training on Columbia undergraduate campus, with the goal of facilitating national dissemination [12,13].
- The CTSA connected Columbia investigators to CTSA-affiliated investigators in other institutions, and an innovative U01 application was submitted to NCATS, which has been funded.
- At a CTSA meeting, an investigator noted a lack of resources to develop cost-effectiveness analyses and was connected to an economist. The collaboration has led to a grant proposal which incorporates cost-effectiveness analysis into the intervention effectiveness study.
- Following an email blast, a surgeon was added to the Advisory Board of a recently funded grant, providing needed expertise to facilitate the research.

**Table 2:** Grant applications from CTSA-affiliated investigators

Number of unique grant applications	41
Number of funded grants to date	14
Number of pending grants to date	17
Number of unfunded grants to date	10
Number of Principal Investigators	20
Schools/Departments of Principal Investigators	Psychiatry Epidemiology Health Policy and Management Sociomedical Sciences Anesthesiology Orthopedic Surgery Vagelos College of Physicians and Surgeons School of Social Work Mailman School of Public Health School of General Studies School of Nursing
Examples of CTSA involvement	Introductions to potential collaborators Individual technical assistance Workgroup participation Executive Committee participation Meeting participation Listserv participation
Selected grant topics	Testing new treatments Pain management Vaccines Opioid alternatives Relapse prevention Naloxone distribution channels Overdose outcomes Neonatal exposure Effects of policy interventions Coordinated opioid use after dental care, surgery, infectious disease

### Columbia’s CTSA Support for Columbia’s New York State HEALing Communities Proposal

Efforts to facilitate interdisciplinary research provided critical support to facilitate the development of the New York State HEALing Communities project, a multistate project funded by the National Institute of Drug Abuse that aims to reduce opioid-related deaths by over 40% in the next 3 years [14]. The New York project is led by Drs. Nabila El-Bassel (Columbia University School for Social Work) and Edward Nunes (Columbia University Department of Psychiatry), with collaboration from investigators in public health and data science, as well as with external academic institutions in the New York City area (Weill-Cornell Medical College, City University of New York, and Einstein College of Medicine) and nationwide (Yale University and University of Miami). Government partners include the NYS Department of Health, NYC Department of Health and Mental Hygiene, NYS Office of Alcoholism and Substance Abuse Services, and multiple County Health and Mental Health Commissioners.

The key investigators were introduced to each other through CTSA opioid crisis brainstorm activities. Drs. Nabila El-Bassel and Edward Nunes, PIs on the New York State proposal, were first introduced and brought together by their participation in the Opioid Crisis Initiative Community Work Group at the brainstorming seminar convened in December 2017. Their discussions in that work group seeded their planning for Columbia’s successful HEALing Communities Study grant application. Their planning efforts were also supported through Irving Institute seed funding directed specifically to address the opioid crisis (below). Moreover, Dr. Frances Levin was introduced to Dr. El-Bassel through CTSA-supported activities, and now the national Substance Abuse and Mental Health Services Administration (SAMHSA) initiatives led by Dr. Levin are components of the awarded HEALing Communities project to support the training initiatives.

The Irving Institute/CTSA helped foster the critically important relationships with semirural/rural upstate counties that are the focus of the HEALing Communities application via a pilot award to Dr. El-Bassel’s group and by supporting Dr. Nunes group’s initiative with Orange County Medical Center. These relationships led to additional grant applications currently under review, in addition to the HEALing Communities project. Moreover, the Irving Institute/CTSA provided introductions and facilitated the research collaborations between Columbia/New York State Psychiatric Institute, Yale University, Medical University of South Carolina and their CTSA hubs as well as other institutions nationally for these innovative applications.

Dr. El-Bassel was a recipient of our Irving Institute inaugural Opioid Strategic Planning pilot award in 2018 for the project entitled, “Mid-Hudson Opioid 2CARE Pilot.” This pilot provided funds to develop and test innovative methods, technologies, and approaches to engage and care for individuals with Opioid Use Disorders in urban and rural communities. This award provided critical proof-of-principle and preliminary data that facilitate a successful HEALing Communities application. The Irving Institute/CTSA also nominated Dr. Timothy Hunt of Columbia School for Social Work, co-investigator on Columbia’s HEALing Communities program, to participate in the NCATS/CTSA Rural Engagement “unmeeting”, University of Florida, Gainesville. The ideas generated and collaborations established at that national brainstorming event, highlighting the disparities that exist and the resources available in cooperative extensions in these communities, will certainly serve our community engagement in New York State and collaborations with other HEALing Communities centers and the national CTSA hub network.

Community engagement and implementation are key to the success of the Columbia HEALing Communities program, and so the work of Columbia’s Opioid Crisis Initiative Community Work Group and the Irving Institute/CTSA Implementation Science Program established in 2018 are providing critical support to the regional and national HEALing Communities program. Columbia’s CTSA and Irving Institute leadership are working directly with Drs. Nabila El-Bassel and Edward Nunes, PIs of Columbia’s HEALing Communities award, to strategize how the Irving Institute/CTSA extensive resources, including its Opioid Crisis Response Initiative, as well as other New York CTSA Program hubs, and the national CTSA Program network, can effectively support community engagements and care model implementation for the regional and national HEALing Communities program.

Thus, the CTSA emphasis on interdisciplinary collaboration across the medical center, and locally and nationally, as well as the development of relationships with state and local government partners and community leaders, has contributed to the emergence of this dynamic opportunity to participate in this national effort to rapidly reduce opioid-related deaths through the development and implementation of evidence-based practices to communities experiencing high rates of opioid deaths.

## Discussion

Columbia’s CTSA Program hub has acted quickly to bring together a large team-science-based, collaborative interdisciplinary initiative, from different divisions across the university, other local academic medical centers and universities, state and local policymakers, and community-based organizations. While investigators at Columbia have an extensive prior track record of innovative substance use research, including opioid research, the activities described in this paper serve as examples of how silos can be bridged to build on existing strengths and facilitate broader interdisciplinary research. These CTSA-initiated activities were designed to enable investigators to mobilize when the NIH HEAL initiative launched. While it is still too soon to identify the public health impacts of this funded research, monitoring outcomes is a component of such research. For example, HEALing Communities cite a goal of reducing opioid-related deaths by 40% in the next 3 years. Columbia CTSA-affiliated investigators, notably Arthur Robin Williams, are examining how best to effectively assess outcomes [15]. After nomination by our initiative leadership, he was appointed to a National Quality Forum committee [16] of diverse experts charged to oversee a review of measures and concepts related to medical opioid use, and opioid use disorder prevention, treatment, and recovery. This activity will further identify measure gaps and priorities relevant to the US opioid overdose epidemic and the broad health care quality challenges that surround it.

Going forward, the CTSA will continue to support team science interventions, through regular large and small group meetings, investigator matchmaking locally and nationally, the provision of core resources and seed funding to help interdisciplinary projects secure grant funding, and the assessment of outcomes to examine the public health impact of opioid-related research.

This effort showcases how a CTSA hub can work to help disparate organizations (within and outside the academic medical center) and disciplines work together to address a significant public health crisis. As new public health crises are identified, the processes developed here (identifying leaders, expanding outreach to the full campus, community partners, national partners, and the general public, and ongoing support for collaboration development) are being used to support interdisciplinary approaches for other research challenges and public health crises. For example, the Columbia CTSA hub has created an Implementation Science Initiative and an Exposomics Initiative, which is applying a similar process – identifying and engaging key experts and leaders to brainstorm effective strategies for campus-wide outreach and ongoing support of collaborations, interdisciplinary teams, and proposal development.

Through their interdisciplinary nature, CTSA hubs are uniquely positioned to help academic medical centers work across disciplines to address public health needs. The model used here is now being used by other CTSA initiatives and can be adopted by the over 50 CTSA hubs nationwide. CTSA leaders should meet regularly and stimulate NIH and NCATS responses, to address new public health crises that may require interdisciplinary interventions, and can begin the process laid out here to stimulate research collaborations.
